# Questionable safety of thyroid surgery with same day discharge

**DOI:** 10.1308/003588412X13373405384576

**Published:** 2012-05

**Authors:** HE Doran, J England, F Palazzo

**Affiliations:** on behalf of the British Association of Endocrine and Thyroid Surgeons

**Keywords:** Thyroid, Thyroidectomy, Day case, Ambulatory, Bleeding, Hematoma

## Abstract

**INTRODUCTION:**

Over the last two decades increasing numbers of surgical procedures have been performed on an outpatient basis. In 2000 the National Health Service in England set the target of performing 75% or more of all elective surgical procedures as day cases and in 2001 the British Association of Day Surgery added thyroidectomy to the list of day case procedures. However, same day discharge following thyroidectomies has been adopted by only a very small number of UK centres. The aim of this review was to establish the evidence base surrounding same day discharge thyroid surgery.

**METHODS:**

The British Association of Endocrine and Thyroid Surgeons commissioned the authors to perform a review of the best available evidence regarding day case thyroid surgery as a part of a consensus position to be adopted by the organisation. A MEDLINE® review of the English medical literature was performed and the relevant articles were collated and reviewed.

**RESULTS:**

There are limited comparative data on day case thyroid surgery. It is feasible and may save individual hospitals the cost of inpatient stay. However, the risk of airway compromising and life threatening post-operative bleeding remains a major concern since it is not possible to positively identify those patients most and least at risk of bleeding after thyroidectomy. It is estimated that half of all post-thyroidectomy bleeds would occur outside of the hospital environment if patients were discharged six hours after surgery.

**CONCLUSIONS:**

Same day discharge in a UK setting cannot be endorsed. Any financial benefits may be outweighed by the exposure of patients to an increased risk of an adverse outcome. Consequently, 23-hour surgery is recommended.

The development of dedicated day case units coupled with improvements in surgical and anaesthetic techniques has led to increasing numbers of surgical procedures being performed on an outpatient/ambulatory basis. The duration of hospitalisation contributes significantly to the total cost of surgical care so the potential cost savings of day surgery are significant and desirable.^1^ In 2000 the National Health Service (NHS) in England set the target of performing 75% or more of all elective surgeries as a day case^2^ and in 2001 the British Association of Day Surgery added thyroidectomy to the list of procedures deemed suitable for the day case environment. Optimal patient and disease selection were not specified and there are currently no published UK day case thyroidectomy guidelines.^3^ Although some selection criteria based on medical and social status, size of the thyroid, extent of thyroidectomy or associated procedures (lobectomy or total thyroidectomy, concomitant lateral neck dissection etc) have been suggested, no consensus exists.^4–6^

The British Association of Endocrine and Thyroid Surgeons (BAETS) is the UK’s leading organisation for surgeons with an interest in thyroid surgery. In 2010 it commissioned the authors to perform an evidence-based review of day case thyroid surgery for presentation and debate at the 2011 annual conference.

## Premise

Day case thyroidectomy is defined as that which does not involve an overnight stay in a fully equipped and staffed hospital ward. This excludes short stay surgery, 23-hour stays, post-operative hotel accommodation adjacent to the hospital with low grade nursing care and other loose adoptions of the terms ambulatory or day care surgery. In the absence of randomised trials, validated series or well constructed relevant systematic reviews were favoured as sources.

For day case thyroid surgery to be advocated in the UK, it needs to be feasible, of demonstrable benefit to the patient and hospital, and advantageous to the publicly funded healthcare system. It should not expose the patient to any additional risk compared with patients staying in hospital and the risk–benefit analysis needs to support the practice.

## Feasibility

There can be little doubt that thyroid surgery can be performed in the majority of cases without the need for hospital admission for longer than is required for pain relief and recovery from anaesthesia. A series of 1,242 consecutive thyroidectomies performed in Texas suggested that a successful day case rate of over 80% can be achieved if patients are selected appropriately.^7^ Inabnet *et al* also reported a day case rate of 80% in 224 cases, citing the use of surgery under local anaesthesia and sedation as well as better haemostatic techniques as the key to success.^8^

In contrast, a study from Georgia, US, reported a series of 418 thyroidectomies, in which only 36% of patients undergoing a total or completion thyroidectomy were discharged on the day of surgery, implying that unilateral surgery is better suited to the day case setting.^5^ Day case thyroid surgery in the US seems to be feasible but publication bias is misleading since it actually represents a very small proportion of all thyroidectomies performed each year. It is also frequently unclear whether the patients are truly discharged home given the variable local geography and mobility of the insured American patient.

Approximately 10,000 thyroidectomies are performed each year in the UK and it can be estimated that, currently, less than 1% are performed as a day case.^9^ As a consequence, insufficient UK-based information is available to establish whether same day discharge is safe in the UK context. Day case surgery in the UK involves discharge home rather than to any other establishment such as a hotel or an intermediate establishment with low level nursing care.

## The challenge of patient selection

A study of 1,571 23-hour (rather than day case) thyroidectomies demonstrated that less than 2% of patients require more than 23 hours in hospital so long as they are undergoing first time neck surgery, are euthyroid, have an ultrasonography estimated thyroid volume of less than 80ml, do not have a locally advanced tumour, a retrosternal/intrathoracic goitre or require a lateral neck dissection.^4^ Less information is available about selection for true day case thyroidectomies and a review published in 2008 failed to identify specific selection criteria.^10^ The generic general health, social and logistical factors of day case surgery are well established. However, anticipating the difficulty of a thyroid procedure and the risk of complications remains less reliable.

Thyroid surgery is associated with a small risk of specific life threatening complications, principally airway obstruction from haemorrhage, laryngeal oedema and tetany from severe hypocalcaemia. For day case surgery to be safe, high risk patients would need to be identifiable and excluded. If post-operative complications do occur, it is essential that they are dealt with safely and that the fact that the patient is not located in the hospital should not add excessive additional risk.

## Benefits of the day case environment

There are recognised generic benefits associated with same day discharge. These include improved patient experience, reduced risk of cancellation and financial savings. Day case thyroid surgery requires patient and carer education with clear written information on discharge protocols that explain the necessary actions that would need to be adopted if complications occur. Opioids and other pain relief associated with nausea and vomiting are best avoided, and non-steroidal drugs (in particular aspirin and clopidogrel) should be stopped pre-operatively to reduce the risk of haematoma formation. The use of local anaesthesia including cervical blocks to reduce pain and nausea and facilitate early discharge has also proved advantageous.^7,11,12^

One of the driving forces behind the promotion of day case surgery is the perceived saving to the UK’s finite-resource public funded healthcare system. However, few studies are available that look specifically at the finances of thyroid surgery in a day case setting. Mowschenson and Hodin demonstrated a cost saving of 30% over the inpatient equivalent in 100 consecutive unselected thyroidectomies and bilateral parathyroid explorations.^13^ Nevertheless, whether such a saving would be present if looking at actual cost rather than charges is unclear. Overall costs are so overwhelmed by the cost of the operation and recovery room time that the differential effect of discharge at 6, 8 or 23 hours appears small.

The report by Marohn and LaCivita on 150 cases of total/near total thyroidectomy with an overnight stay using a crude cost analysis based on $1,000/day for military and $2,000/day for civilian average bed costs gave a saving of $1,540 and $3,080 per patient respectively for an average length of stay of 1.06 days compared with an average pre-authorised length of stay of 2.6 days (ie net bed day saving 1.54 per patient).^14^ Seybt’s report of 418 thyroid procedures where 80% were in hospital for less than 24 hours showed a cost of $7,814 for ambulatory patients compared with $10,288 for inpatients.^5^

In a UK context the saving of one night in patient stay accounts for a cost saving of £350–£430 per night. Based on a Health Resource Group remuneration of just under £3,000 for a thyroidectomy, this saving represents over 10% and may mean the difference between profitability and loss in some centres. Potential further savings are available by the use of local anaesthesia.^11,15^

## Patient satisfaction

Patients’ preference for same day discharge has been demonstrated generically.^16^ Whether this applies to a fully informed thyroidectomy patient is less clear. Mowschenson and Hodin looked at day case patient preference within their overall series, comparing this with a control group of 30 day case laparoscopic cholecystectomy patients.^13^ Thirty-five and thirty-two per cent respectively stated they would have preferred an inpatient stay. However, nine patients in the thyroidectomy group were in the inpatient group because of a specific preference so the proportion preferring a hospital admission is probably higher. Samson *et al’*s study of 655 of 869 patients undergoing outpatient thyroidectomy (OPT) compared to standard thyroidectomy (ST) showed that almost all patients undergoing OPT were ‘very pleased’ compared with those with ST stating they were just ‘pleased’ (*p*<0.001).^17^ Spanknebel *et al* also found that very few patients expressed dissatisfaction with their experience of OPT.^11^

The weight of such findings is, however, debatable since satisfaction is a vague retrospective concept that may be used mistakenly as a surrogate for patient preference. Only a blinded, anonymised questionnaire to patients before and after discharge for both inpatient and day case procedures that includes pain scores, satisfaction scores, willingness to repeat and whether they would recommend the procedure as a day case will clarify whether day case thyroidectomy is preferable. The patient must be informed explicitly that there is a very small risk of a life threatening complication at home if discharged on the day of surgery since a small unpublished pilot study by one author (FP) suggests that this, perhaps unsurprisingly, affects patient preference dramatically towards an inpatient stay.

## Post-operative hypocalcaemia

The incidence of clinically significant hypocalcaemia following a hemithyroidectomy is negligible and is therefore not a contraindication to day case single lobe thyroid surgery. Known risk factors for hypocalcaemia following thyroid surgery include total rather than hemithyroidectomy, a raised pre-operative thyroxine level, thyroid cancer and substernal thyroid extension.^18^ For total thyroidectomy patients, the availability of improved methods of prediction of hypocalcaemia^19,21^ and improved understanding of other contributory factors (for example vitamin D deficiency) mean that clinically significant hypocalcaemia can usually be obviated.

National audit data demonstrate that up to a third of patients undergoing a total thyroidectomy^20,21^ may become hypocalcaemic and require calcium and/or vitamin D analogue supplements. Readmission rates for hypocalcaemia should, however, be less than 2% if treated appropriately.^7^ The routine use of prophylactic calcium is controversial^22^ but it is often used in short stay thyroid surgery. Nevertheless, giving routine calcium and vitamin D analogues is not without risk since hypercalcaemia may occur with potentially serious deleterious effects on renal function.^23,24^

## Laryngeal nerve palsy

Recurrent laryngeal nerve (RLN) paralysis is a well recognised complication of thyroid surgery. Routine post-operative laryngoscopy has demonstrated that a degree of temporary reduced vocal cord mobility following thyroid surgery affects up to 10% of patients.^25^ This is most frequently caused by RLN injury but, rarely, vocal cord paralysis may also be caused by arytenoid cartilage subluxation, cricoarytenoid arthritis or neoplasm. The most common cause of reduced vocal cord mobility following thyroid surgery, however, is RLN neurapraxia, from which the patient should recover within days or weeks. The incidence of permanent RLN injury should be under 1–2%^26,27^ although anonymised national databases where routine laryngoscopy has been used show a significantly higher rate.^28^ The risk of permanent laryngeal nerve injury in revision thyroid surgery is approximately six times higher than in first time thyroid surgery.^20^

For day case thyroidectomy to be safe and acceptable, the benefit of same day discharge should outweigh the risk of airway embarrassment due to RLN injury. If a patient has been appropriately prepared, a unilateral nerve paralysis should not normally result in inpatient conversion. Only very rarely can a unilateral nerve injury result in airway embarrassment, usually due to vocal fold synkinesis, which may occur months after nerve injury.^29^

Bilateral RLN paralysis is uncommon. Its incidence is difficult to establish but it has been reported as 0.38% (1 in 263 cases) in one series^30^ and 0.2% (1 in 500) in Sweden’s national thyroid and parathyroid surgery registry.^27^ As its occurrence constitutes an anaesthetic emergency, this would necessitate the patient to be admitted. Due to its relative rarity, the risk of bilateral paralysis should not in itself mitigate against advocating day case bilateral thyroid surgery and can be minimised further by patient selection with the avoidance of high risk cases, such as bilateral revision thyroid surgery, or in cases with a pre-existing unilateral palsy.

## Post-operative bleeding

Post-operative haemorrhage is the single biggest concern in day case thyroid surgery. It occurs in between 0.9%^20,26,31–33^ and 2.1%^27^ of all thyroidectomies and may be a life threatening event due to local compression and laryngeal oedema. During a 40-year period over which 10,201 thyroidectomies were performed at the Royal North Shore Hospital in Sydney, 124 (1.2%) required reoperation for haemorrhage with 31 (0.3%) also requiring a tracheostomy.^33^

Thirty-day mortality following thyroid surgery in the UK is 1 in 500.^20^ An unspecified proportion of these deaths will be secondary to a post-operative haemorrhage. A post-operative haemorrhage in the neck after thyroid surgery needs immediate management and at least a quarter require immediate bedside intervention.^31–34^ Presumably as a result of publication bias, there are no reliable data on survival following acute haematoma formation in a day case setting. While it is likely that a post-thyroidectomy haemorrhage at home would increase the mortality risk, this is unproven. It is also unclear whether the bleed after a hemithyroidectomy is less life threatening than after a total thyroidectomy. In the absence of hard data on these issues, the next best thing would be a reliable form of risk stratification that allows the identification of the patients with a minimal bleed risk.

In Burkey *et al’*s retrospective review (1976–2000) of 7,921 thyroidectomies and 5,896 parathyroidectomies, with 21 (0.26%) and 21 (0.36%) post-operative haematomas respectively where a comparison with (non-haematoma) case-matched controls was performed, there were no reliably identified predictive patient or disease related criteria.^34^ Nine of the forty-two patients (21%) who experienced a haemorrhage required an urgent lifesaving bedside decompression and/or intubation.

Others have supported Burkey *et al’*s findings that the extent of thyroidectomy, hyperthyroidism, thyroid resection for malignancy and reoperative surgery do not reliably allow a prediction of the patients most at risk of developing a life threatening haematoma.^27,31,32^ One study did reveal a higher incidence of haematomas requiring evacuation in thyroid reoperations compared with primary procedures and also in reoperative hyperthyroid patients compared with euthyroid patients.^35^ Swedish registry data suggest that older age and male sex are risk factors for bleeding^27^ but a reliable quantification is not possible. Although there are no definitive studies directly comparing new haemostatic technologies, no increase in the incidence of complications has been demonstrated with their widespread adoption.^8,12^ By implication, the reverse is also true.

Given the inability to reliably predict which patients are most or least at risk of a haematoma and with a bleed risk that seems to always be in the order of 1–2%^6–8,13,14,17,20,26,27,31–33,36–39^ the ability to deal safely with the 1 patient in 100 that will bleed following thyroid surgery is paramount. In the context of day surgery, it is clearly the timing and severity of the bleed that is most important. Early bleeds that are recognised and dealt with before discharge are no different to the standard patient treated as an inpatient as long as theatre facilities and staff are readily available to deal with the problem. The extent or severity of a bleed following hemithyroidectomies and total thyroidectomies have not been compared in a systematic way and opinions vary.

Similarly, the perception that early bleeds are more dangerous than later bleeds is anecdotal rather than proven. Mirnezami *et al*’s review suggests that patients with significant haemorrhage display signs of bleeding within the first few hours and those with potential airway obstruction within four hours.^40^ However, this is not supported by large single centre series. A retrospective review of 6,830 thyroidectomies performed in Poitiers, France, reporting 70 haematomas (1%) showed only 37 (53%) occurred within 6 hours (ie when the day care patient would still be hospitalised).^31^ The rest occurred after 6 hours: 26 (37%) between 7 and 24 hours from surgery and 7 (10%) after 24 hours. These findings mirror those of Burkey *et al*, who identified only 43% occurring within 6 hours, 37% between 7 and 24 hours and 19% over 24 hours.^34^ For this reason, these authors have argued that true day case thyroidectomy with discharge within six hours of surgery cannot be considered safe.

Lo Gerfo *et al* countered that retrospective reviews fail to consider sentinel symptoms and that early intervention in such patients may deal immediately with those with a potential for obstruction.^22,37^ Using decision model analysis on earlier US thyroidectomy mortality data, they estimated that 94 haemorrhage related deaths per 100,000 could be prevented by observation for 24 hours (ie advocating a 23-hour stay) as opposed to 6 hours.^22^ Dralle’s observation that 80% of bleeding occurs during the first 24 hours (40% in the first 8 hours) would also appear to support 23-hour stay as the optimal compromise.^12^

## Litigation costs and ascertainment of mortality

Litigation costs (costs of the claim, ie the damages paid to a claimant and legal costs of dealing with the claim) to the NHS following a death/hypoxic brain injury from airway obstruction as a result of post-operative haemorrhage following a thyroidectomy are potentially enormous. The exact cost varies hugely between specific cases (personal communication, NHS Litigation Authority [NHSLA]). Damages awarded depend on the severity of the brain damage, life expectancy, loss of earnings, accommodation and care required if brain damaged as well as the number and age of dependants. If 24-hour care is required, a settlement of £2 million or more is common. Legal costs of around £250,000 for the claimant and £125,000 for the defence are typical and must be added to the overall cost.

Settlements following a death are usually much lower than those for causing a hypoxic brain injury since the bereavement award is usually just £11,800, to which are added loss of lifetime earnings, loss of dependency and funeral expenses etc. The average claim for the death of a 45-year-old man is currently approximately £400,000 (NHSLA). One excess death or major injury following a day case thyroidectomy would eliminate the financial benefit from almost a 1,000 saved night stays achieved due to day case thyroid surgery.

## Conclusions

Day case thyroid surgery is feasible but the most compelling criticism is the threat of post-operative bleeding with a potentially devastating effect on the airway. No other operation performed currently as a day case is associated with a recognised predictable and quantifiable complication that may lead to airway compromise and potentially death within 30 minutes.

Robust generic and disease specific criteria to guarantee safety with respect to the incidence and timing of life threatening haemorrhage are unidentifiable. Evaluating patients suitable for discharge six hours after surgery and retaining those where there is concern would add safety but this does not currently appear possible. Half of post-thyroidectomy bleeds occur after six hours, probably in the absence of symptoms or signs at the time of discharge. Life threatening bleeds appear to occur in an apparently random way. Day case thyroidectomy may save the hospital several hundred pounds per patient but the potential cost of an adverse event is such that any savings accumulated over years of day case surgery would be eliminated by a single significant bleed at home.

The risk of bleeding reduces with hours passed and nearly all significant bleeds occur within 24 hours of surgery. This supports short stay rather than day care thyroidectomies. Increased surgeon volume and experience with thyroidectomies has been shown to give improved outcomes^41^ and may also represent a focus for risk management in the future. However, at present, following review of the available evidence, the BAETS cannot currently endorse thyroidectomy with same day discharge. Focus is required on shortening the current typical post-thyroidectomy length of stay to ideally one post-operative day.
